# Monolayer PdSe_2_: A promising two-dimensional thermoelectric material

**DOI:** 10.1038/s41598-018-20918-9

**Published:** 2018-02-09

**Authors:** Dan Qin, Peng Yan, Guangqian Ding, Xujin Ge, Hongyue Song, Guoying Gao

**Affiliations:** 1 Binzhou Medical University, Yantai, Shandong 264003 P. R. China; 2 School of Physics, Huazhong University of Science and Technology, Wuhan, Hubei 430074 P. R. China; 3 Chongqing University of Posts and Telecommunications, Chongqing, 400053 P. R. China

## Abstract

Motivated by the recent experimental synthesis of two-dimensional semiconducting film PdSe_2_, we investigate the electronic and thermal transport properties of PdSe_2_ monolayer by using the density functional theory and semiclassical Boltzmann transport equation. The calculated results reveal anisotropic transport properties. Low lattice thermal conductivity about 3 Wm^−1^ K ^−1^ (300K) along the *x* direction is obtained, and the dimensionless thermoelectric figure of merit can reach 1.1 along the *x* direction for *p*-type doping at room temperature, indicating the promising thermoelectric performance of monolayer PdSe_2_.

## Introduction

Thermoelectric materials, which enable a direct conversion between heat and electricity via either Seebeck or Peltier effect, have attracted much attention as a sustainable energy resource in the last decade^1^. The conversion efficiency of a thermoelectric material is quantified by the dimensionless thermoelectric figure of merit (*ZT*), which is defined as *ZT* = *S*^2^*σT*/(*κ*_*e*_ + *κ*_*l*_), where *S* is the Seebeck coefficient, *σ* the electrical conductivity, *T* the absolute temperature, *κ*_*e*_ and *κ*_*l*_ the electronic and lattice thermal conductivities, respectively. Obviously, higher power factor (*PF* = *S*^2^*σ*) and lower thermal conductivity are beneficial for improving the thermoelectric performance. The all-scale electronic and atomistic structural engineering techniques have been used to enhance *ZT* values to 2 within a temperature range of 700 ~ 900 K^2–5^. Another promising simple structures exhibit intrinsically low thermal conductances without requiring sophisticate structural engineering such as SnSe crystal and with *ZT* value of 2.6 at 923 K^6^, although this value falls quickly for lower temperatures.

Since the discovery of graphene in 2004^7,8^, many 2D structures of inorganic layered materials, such as black phosphorus^9–11^ and h-BN^12,13^ etc., have been experimentally realized during the last decade. It has been proposed that low-dimensional materials could have better thermoelectric performance than their bulk due to the diverse scattering mechanism for phonons and intrinsic energy dependence of their electronic density of states^14–16^. And even in high dimensional materials, one can make use of the effective low dimensionality of the electron band to increase the thermoelectric performance^17–19^. Recently, the class of transition metal dichalcogenide (TMD) with one layer of transition metal sandwiched between two layers of chalcogen atoms have been a subject of extensive studies due to their fantastic electronic properties^20–22^. However, the *ZT* values of 2H- MoSe_2_, MoS_2_ and WSe_2_ monolayers are about 0.1 at 1200 K^23^, 0.11 at 500 K^24^ and 0.7 at high temperature^23^, respectively. It was confirmed that such a low *ZT* is mainly caused by a high lattice thermal conductivity *κ*_*l*_. While those with CdI _2_ type typically represented by *M* = Ti, Zr, Hf, etc. have much lower lattice thermal conductivities. For example, the *κ*_*l*_ values of monolayer ZrSe_2_ and HfSe_2_ are 1.2 and 1.8 Wm^−1^ K^−1^ ^25^, respectively at 300 K, leading to optimum *ZT* values of 0.87 and 0.95, respectively.

Most recently, another class of layered materials formed by noble metals, such as Pt and Pd, with S and Se atoms have been investigated both experimentally and theoretically^26–30^. Importantly, the monolayer PdSe_2_ has very recently been exfoliated from bulk crystals by Akinola D. Oyedele *et al*.^28^, which is a pentagonal 2D layered noble transition metal dichalcogenide with a puckered morphology that is air-stable. The experimental results by Oyedele *et al*. demonstrated that few-layer PdSe_2_ displayed tunable ambipolar charge carrier conduction with a high electron apparent field-effect mobility of ~158 cm^2^ V^−1^ s^−1^. In addition, the puckered 2D PdSe_2_ flakes exhibit a widely tunable band gap that varies from metallic (bulk) to ~1.3 eV (monolayer). Motivated by this, we expand our knowledge on the thermoelectric properties on the monolayer PdSe_2_ in this work. And to the best of our knowledge, there is no utter investigation in the thermoelectric properties of the monolayer PdSe_2_. In this paper, we investigate PdSe_2_ monolayer with the configuration of the above experiment, performing electronic structure, and phononic transport calculations based on density functional theory (DFT) and Boltzmann transport theory. The results show that monolayer PdSe_2_ is an indirect semiconductor, with a band-gap value of 1.38 eV, which is in good agreement with ref.^28^. Based on the electronic and phononic properties, we study the thermoelectric properties of monolayer PdSe_2_. We obtain the Seebeck coefficients for monolayer PdSe_2_ and a maximum *p*-type figure of merit, 1.1, along the *x* direction at the optimal doping (300 K). We also find anisotropic characters in electrical conductivity and thermal conductivity which are derived from the asymmetric structure of the monolayer PdSe_2_ in plane.

## Results and Discussions

### Geometric structure

**Figure 1:**
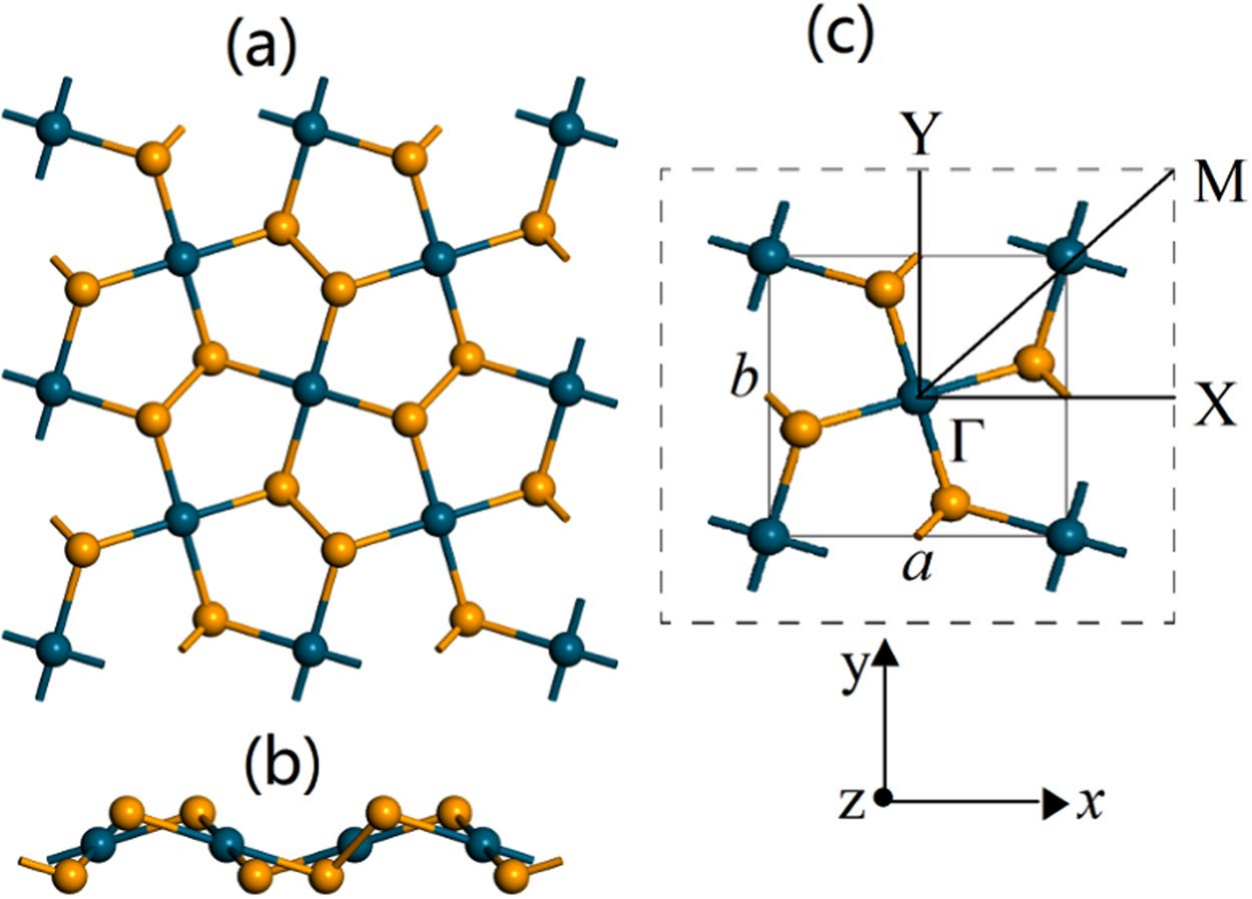
(**a**) and (**b**) are the top and side views of PdSe_2_ monolayer, respectively. (**c**) The unit cell and corresponding Brillouin zone path with the high-symmetry points at Γ(0, 0, 0), X(0.5, 0, 0), M(0.5, 0.5, 0) and Y(0, 0.5, 0). The lattice parameters are denoted as *a* and *b*, which are along the *x* and the *y* directions, respectively. Cyan: Pd atom. Yellow:Se atom.

In our calculations, the monolayer structure is obtained from the experimental bulk structure PdSe_2_ with *a* = 5.75 *Å*, *b* = 5.87 *Å*, and *c* = 7.69 *Å*^31^. The monolayer PdSe_2_ is cut through the (0 0 1) plane of the PdSe_2_ crystal, and a vacuum slab about 21 Å is added in the direction perpendicular to the nano-sheet plane (*z* direction). As shown by the side view and projected top view of the PdSe_2_ monolayer in Fig. 1(a) and (b), each Pd atom binds to four Se atoms in the same layer, two neighboring Se atoms can form a covalent Se-Se bond^32^ and two Pd atoms and three S atoms can form a wrinkled pentagon, which is rather rare in known materials. In addition, we note that the space group has changed from *pbca* to *pca*2_1_ evolving from bulk to monolayer, which has been found in experiments^27^. The unit cell of monolayer PdSe_2_ is displayed in Fig. 1(c) and the optimized lattice parameters of monolayer PdSe_2_ are *a* = 5.7538 *Å* and *b* = 5.9257 *Å*, which are in good agreetment with the previous reports^26,27^.

**Figure 2:**
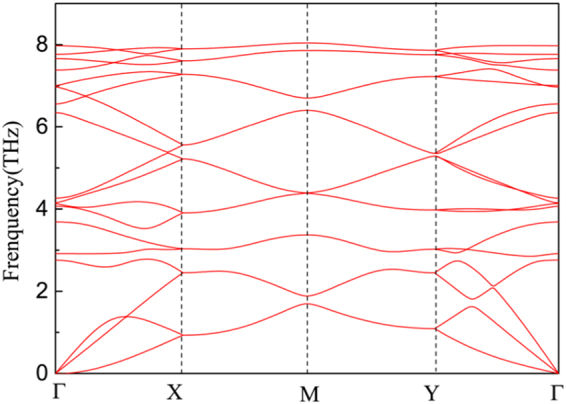
Calculated phonon dispersion spetrum of monolayer PdSe_2_. Here the band structure is along the high-symmetry points at Γ(0, 0, 0), X(0.5, 0, 0), M(0.5, 0.5, 0), Y(0, 0.5, 0) and Γ(0, 0, 0).

In order to verify the stability of the monolayer PdSe_2_, we perform phonon dispersion calculations^33^. As represented in Fig. 2, there are no soft modes in the calculated phonon dispersions, indicating the dynamical stability of this structure. This is also consistent with the previous reports^28,31^.

### Electronic transport properties

**Figure 3:**
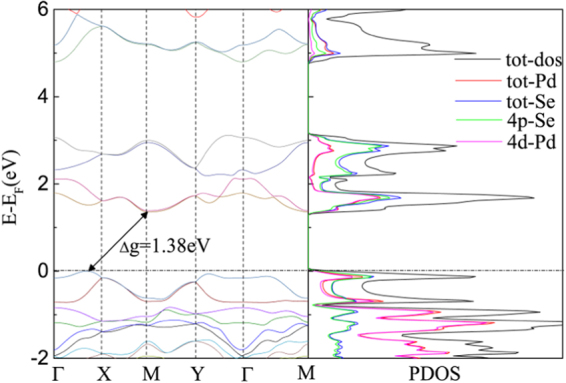
Calculated electronic band structure (left) and density of states (right) of monolayer PdSe_2_ with indirect band gap of 1.38 eV. Here the band structure is along the high-symmetry points at Γ(0, 0, 0), X(0.5, 0, 0), M(0.5, 0.5, 0), Y(0, 0, 0.5) and Γ(0, 0, 0). The solid arrows indicate the lowest energy transitions between the valence band maximum (VBM) and conduction band minimum (CBM).

Experimental and theoretical studies have demonstrated that monolayer PdSe_2_ exhibits high mobility and Seebeck coefficient^26,27^, which are beneficial for the thermoelectric transport. Now we first turn to the investigation of electronic transport properties. Based on the above-determined configuration, we calculate the electronic band structure with the Brillouin zone path along Γ − *X* − *M* − *Y* − Γ as shown in Fig. 1(c). Computed via the TB-mBJ-GGA potential with spin-orbit coupling (SOC) included, the PdSe_2_ monolayer is semiconducting with an indirect band gap of 1.38 eV, which is in general agreement with the previous reports^26,34^, as depicted in Fig. 3. The conduction band minimum (CBM) locates at the M (0.5, 0.5, 0) points, while the valence band maximum (VBM) locates in the interval between Γ and X (0.5, 0, 0) points. The projected density of states reveals that the *d*-states of the transition metal atoms and *p*-states of the selenium atoms contribute most to the states at both VBM and CBM.

The effective mass m^*^ near the Fermi energy is an important parameter for the thermoelectric transport^35^, which can be extracted from the high-precise energy band calculation via the equation

1 $$\frac{1}{{m}_{\alpha }^{\ast }}=\frac{1}{\hslash }\frac{{\partial }^{2}E({k}_{a})}{{\partial }^{2}{k}_{\alpha }}$$

where *ћ* is the reduced Plank’s constant, *E*(*k*_*α*_) is the band index *α* and wave vector k dependent energy. Thus, on the basis of the electronic band calculations, we can obtain the effective m* of electrons and holes in the *x* and *y* directions. As listed in Table 1, the effective mass along Γ-X and Γ-Y are 0.30(e), −0.25(h) and 0.12(e), −0.16(h), respectively. Obviously, in the *m*_*e*_ unit of free electron mass, the effective masses along Γ-X are significantly larger than that along Γ-Y direction and even in the same direction there are slightly differences between holes and electrons, indicating the anisotropic electronic properties of monolayer PdSe_2_. Besides the band gap and effective mass, carrier mobility is another important factor for semiconducting materials in electronic transport properties. Therefore, in order to obtain more information on the transport properties of monolayer PdSe_2_, we investigate its carrier mobilities on the basis of Bardeen-Shockley deformation potential (DP) theory in 2D materials^36,37^. Note that the DP theory has been successfully performed to present the carrier mobility of many 2D structures^38–41^. Although the results may be less accurate, it can still reflect the basic and general thermoelectric performance of materials. According to the DP theory, the carrier mobility (*μ*) of 2D structure can be expressed as

2 $$\mu =\frac{e{\hslash }^{3}{C}_{2D}}{{k}_{B}T{m}^{\ast }{m}_{d}{E}_{l}^{2}}$$

where *k*_*B*_ is the Boltzmann constant, *T* is the temperature, *m*_*d*_ is the average effective mass defined as ${m}_{d}=\sqrt{{m}_{x}^{\ast }{m}_{y}^{\ast }}$ (${m}_{x}^{\ast }$ and ${m}_{y}^{\ast }$ are the effective mass along the *x* and *y* directions, respectively). *C*_2*D*_ is the in-plane effective elastic modulus for 2D system defined as ${C}_{2D}={\frac{1}{{S}_{0}}\frac{{\partial }^{2}E}{\partial {(l/{l}_{0})}^{2}}|}_{l={l}_{0}}$, where *E* and *l* are the total energy and lattice constant after deformation, *l*_0_ and *S*_0_ are the lattice constant and cell area at equilibrium for 2D system. *E*_*l*_ is the deformation potential constant determined by ${E}_{l}={\frac{\partial {E}_{edge}}{\partial (l/{l}_{0})}|}_{l={l}_{0}}$, where *E*_*edge*_ is the energy value of CBM (for electrons) and VBM (for holes). All the results are summarized in Table 1. The in-plane effective elastic modulus is 1.92 (*x* direction) and 1.17 (*y* direction) eV/*Å*^2^ much lower than those of MoS_2_ (7.99 eV/*Å*^2^)^39^ and PdS_2_ (3.62 eV/*Å*^2^ in the *x* direction and 5.11 eV/*Å*^2^ in the *y* direction)^30^, indicating that PdSe_2_ is much softer than MoS_2_ and PdS_2_ monolayer. As have been investigated in previous works, such large flexible deformation may improve the electronic properties via the compression (tensile) strain^29,42–44^. By fitting the band edge-strain curves, we find that the deformation potentia_*l*_s (E _*l*_) of holes are rather small, namely −2.61 (*x* direction) and −2.89 (*y* direction), compared with the values of electrons of −8.49 (*x* direction) and −9.11 (*y* direction) cm^2^ V^−1^ s^−1^, respectively. Deformation potential constants describe the scattering caused by electron-acoustic phonon interactions. Thus, small of deformation potential constants may lead to large carrier mobilities. Then, based on the Equation 2, the acoustic phonon-limited carrier mobilities have been estimated. As shown in Table 1, the mobilities of electrons are 159.92 and 211.59 cm^2^ V^−1^ s^−1^ in the *x* and *y* directions, respectively. Whereas the mobilities of holes are 1928.99 (*x*) and 1498.03 (*y*), which are much larger than those of electrons mainly due to the rather small E_*l*_. However, the mobilities of both holes and electrons for the PdSe_2_ monolayer are larger than those of the MoS_2_^39^ and PdS_2_^30^, indicating that the monolayer PdSe_2_ would be a quite promising material for electronic and thermoelectric applications.

**Table 1:** The computed effective mass (m*), average effective mass (*m*_*d*_), elastic modulus *C*_2*D*_, DP constant *E*_*l*_, carrier mobility (*μ*), relaxation time (*τ*) of electrons and holes along the *x* and *y* directions for the PdSe_2_ monolayer at 300 K.

directions	carriers	m* (*m*_*e*_)	*m*_*d*_ (*m*_*e*_)	*C*_2*D*_ (eV/*A*^2^)	*E*_*l*_(eV)	*μ* (*cm*^2^ *V*^−1^ *s*^−1^)	*τ* (10^−14^ *s*)
*x*	e	0.30	0.19	1.92	−8.49	159.92	2.73
	h	−0.25	0.20	1.92	−2.61	1928.99	27.46
*y*	e	0.12	0.19	1.17	−9.11	211.59	1.44
	h	−0.16	0.20	1.17	−2.89	1498.03	13.64

**Figure 4:**
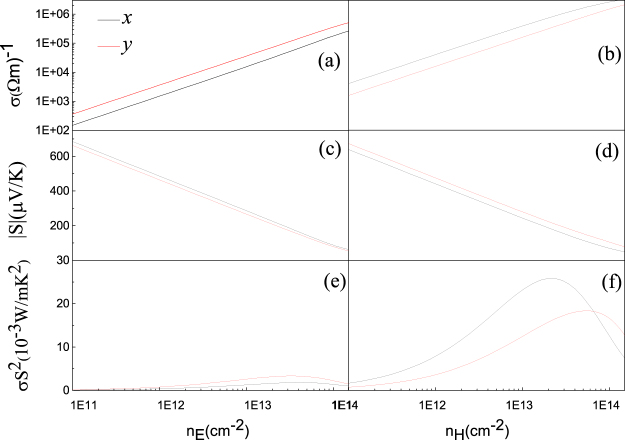
Calculated thermoelectric transport coefficients *σ* (**a**,**b**), *S* (**c**,**d**), and *S*^2^*σ* (**e**,**f**) vs carrier concentration for *n*-(left panels) and *p*-type (right panels) doped PdSe_2_ along the *x* (black lines) and the *y* (red lines) directions at room temperature.

Now we are in a position to evaluate the electronic transport coefficients such as Seebeck coefficient *S* and electrical conductivity *σ*, based on the CRTA Boltzmann theory. The left (right) panels of Fig. 4 show the transport coefficients along the *x* and *y* directions as a function of the electron (hole) concentration at *T* = 300 K. It is clear that the *σ* in Fig. 4(a,b) increases with the increasing carrier concentration while the magnitude of *S* in Fig. 4(c,d) decreases with doping. The electrical conductivity *σ* of monolayer PdSe_2_ exhibits remarkable anisotropic behaviors with (*σ*_*y*_/*σ*_*x*_) ~2.3 for *n*-type doping and (*σ*_*x*_/*σ*_*y*_) ~2.4 for *p*-type at 1.1 × 10^13^ cm^−2^ concentration. The calculated Seebeck coefficients along the *x* and *y* directions as a function of carrier concentration are shown in Fig. 4(c) and (d) for *n*- and *p*-type doping, respectively. We find a larger asymmetry of the Seebeck coefficient for *p*-type doping than for *n*-type doping, which is in good agreement with the recent report^26^. This anisotropy in the thermopower values in the two different directions might enable to design transverse thermoelctric device^45^. It is important to note that the Seebeck coefficients for both *n*- and *p*-type doped monolayer PdSe_2_ are substantially high at room temperature, reaching a peak value of 660 *μV*/*K* at an electron concentration around 1.25 × 10^11^ cm^−2^ and with an average value in the range of 300–340 *μV*/*K*. These values of *S* for monolayer PdSe_2_ compare favorably with those reported for some other 2D materials^30,39^. Figure 4(e) and (f) depict the power factor (PF) *S*^2^*σ* at room temperature along the *x* and *y* directions for *n*- and *p*-doped PdSe_2_ monolayer, respectively. The results reflect significant anisotropy in the power factor with the PF_*x*_/PF_*y*_ ~1.9 for *p*-type doping and (PF)_*y*_/(PF)_*x*_ ~2 for *n*-type doping at concentration around 1.1 × 10^13^ cm^−2^. The anisotropy in power factor arises from the large anistotropy of the conductivity and Seebeck coefficient for *p* and *n* types, as described above.

### Phononic transport

Figure 2 shows the phonon dispersion relations of monolayer PdSe_2_ at its equilibrium volume along the high symmetric Γ − *Y* − *M* − *X* − Γ directions. It is noteworthy that the phonon spectrums of monolayer PdSe_2_ is very distinct from the MoS_2_ type monolayer. The maximum frequency of the acoustic mode markedly drop to rather low value of 3.7 THz, while for monolayers of MoSe_2_ and WSe_2_ it is 5.4 THz and 4.8 THz, respectively, and even higher for monolayer MoS_2_ with the value of 7.5 THz. Such low frequency suggests the low group velocity of acoustic modes in monolayer PdSe_2_. As acoustic modes contribute mostly to the lattice thermal conductivity *κ*_*l*_, lower *κ*_*l*_ in this PdSe_2_ monolayer is expected.

**Figure 5:**
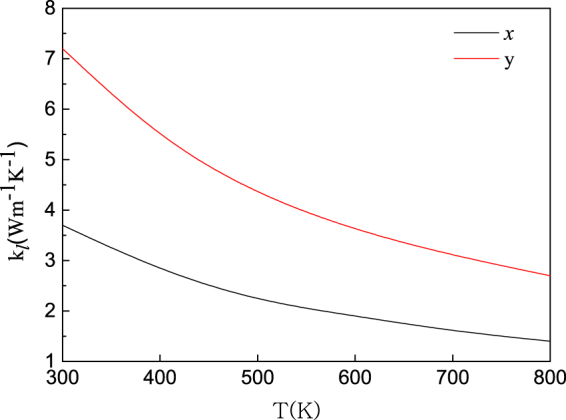
Calculated lattice thermal conductivity of monolayer PdSe_2_ along the *x* (red solid line) and the *y* (black solid line) directions from 300 K to 800 K with the interval of 100 K.

Now we turn to the computation of lattice thermal conductivity *κ*_*l*_. As mentioned above, we estimate *κ*_*l*_ by means of the phonon Boltzmann transport equation and DFT as implemented in VASP and ShengBTE code. As presented by the fitted lines in Fig. 5, *κ*_*l*_ decreases following a *T*^−1^ dependence with the increasing temperature, suggesting that Umklapp phonon scattering dominates three-phonon interactions^46^. From the calculations, the obtained lattice thermal conductivity of monolayer PdSe_2_ is 3.7 (1.4) and 7.2 (2.7) Wm^−1^ K^−1^ at 300 K (800 K) along the *x* and *y* directions, respectively, which are much lower than MoS_2_^47^ and GX_2_ monolayers^48^. It is obvious that the lattice thermal conductivity exhibits large directional anisotropy which may be due to differences in group velocity, anharmonicity and scattering phase space along the different directions.

### Dimensionless figure of merit *ZT*

The electronic thermal conductivity *κ*_*e*_ of monolayer PdSe_2_ is calculated via the Wiedemann-Franz law *κ*_*e*_ = *L* *σT*. Within the relaxation time approximation, the Seebeck coefficient can be calculated independently of the relaxation time *τ*, but evaluation of the electrical conductivity requires knowledge of *τ*. Here we take into account only the intrinsic scattering mechanism, namely, the interaction of electrons with acoustic phonons. Then the relaxation time *τ* can be evaluated from the equation *τ* = μm*/*e*, here the carrier mobility *μ* and effective mass m* have been calculated in subsection of Electronic transport properties, as listed in Table 1.

**Figure 6:**
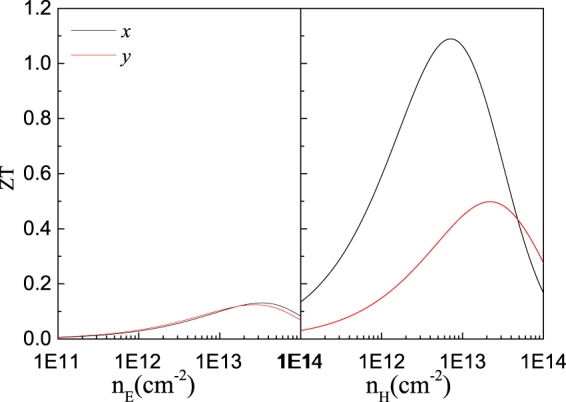
Calculated thermoelectric figure of merit (*ZT*) of monolayer PdSe_2_ with *n*-type doping (left plane) and *p*-type doping (right plane) at 300 K along the *x* (black solid lines) and *y* (red solid lines) directions.

Combining the electronic and thermal transport properties, we now evaluate the thermoelectric performance of the PdSe_2_ monolayer. Figure 6 shows the figure of merit *ZT* value for both *n* and *p* doped PdSe_2_ monolayer along the *x* and *y* directions as a function of the carrier concentration at room temperature. We can see that the *ZT* values of *n*-type doped monolayer PdSe_2_ are rather small and almost isotropic with the maximum value of 0.13 with the corresponding concentration 3 × 10^13^ cm^−2^. However, for *p*-type doped monolayer PdSe_2_, *ZT* values exhibit the strong anisotropic property, with the value along the *x* direction being much larger than that along the *y* direction. The largest *ZT* value of 1.1 can be obtained in the *x* direction at the carrier concentration of 6.5 × 10^12^ cm^−2^ and 0.5 along the *y* direction at the carrier concentration of 2 × 10^13^ cm^−2^, respectively. Therefore, heavily doped *p*-type PdSe_2_ may offer excellent thermoelectric performance for applications such as powergeneration. It is worthwhile to note that we have not considered the thermoelectric performance at higher temperature since the ZA mode of PdSe_2_ monolayer is very soft near point Γ, hence, it may be difficult to remain stable at high temperature. Usually, the thermoelectric performance at room temperature is the most importantly information we need for it is better to discover thermoelectric materials working under room temperature.

## Conclusion

In summary, by means of first-principles calculation, the geometrical structure, mechanical, electronic and thermal transport properties of monolayer PdSe_2_ are systematically investigated. In contrast to TMCs, monolayer PdSe_2_ has strong anisotropic mechanical, electronic and thermal transport properties, leading to anisotropic thermoelectric properties. We find that PdSe_2_ is a semiconductor with an indirect band gap of 1.38 eV and a hole mobility as high as 1929 cm ^2^ V^−1^ s^−1^. The in-plane effective elastic modulus are rather low, suggesting the flexible mechanical properties in this structure. Furthermore, monolayer PdSe_2_ has a low lattice thermal conductivity about 3 Wm^−1^ K^−1^ along the *x* direction at room temperature. Combining its high Seebeck coefficient and markedly low thermal conductivity, monolayer PdSe_2_ shows an optimum *ZT* value of 1.1 (300K) at optimal doping. Therefore, our results indicate monolayer PdSe_2_ is a material with promising thermoelectric performance.

## Computational Methods

The initial structure of monolayer PdSe_2_ is optimized through DFT with the plane-wave based Vienna ab-initio simulation package (VASP)^49,50^, using the projector augmented wave (PAW) method. For the exchange-correlation functional, we have used the Perdew-Burke-Ernzerhof version of the generalized gradient approximation (GGA)^51^. A plane-wave cutoff energy of 400 eV and an energy convergence criterion of 10^−7^ eV are adopted throughout calculations. The spin-orbit coupling (SOC) is not considered in the structure relaxation. For ionic relaxation calculations, a 11 × 11 × 1 Monkhorst-Pack k-meshes^52^ are used and the structure is considered to be stable when the Hellmann-Feynman forces are smaller than 0.001 eV/Å. For the slab model, a 21 Å thick vacuum layer was used to avoid the interactions between adjacent monolayers.

After determining the equilibrium structure, we have performed electronic structure calculations employing the all-electron full-potential WIEN2k code^53^ using recently implemented Tran and Blaha’s modified Becke-Johnson (TB-mBJ)^54^ exchange potential plus generalised gradient approximation (GGA) with the SOC included. The TB-mBJGGA potential for electronic properties and band gap with higher accuracy and less computational effort as compared to hybrid functional and GW overcomes the shortcoming of underestimation of energy gap in both LDA and GGA approximations^55^. The number of plane waves in a Fourier expansion of potential in the interstitial region was restricted to *R*_*MT*_ × *K*_*max*_ = 8. The muffin tin radii for Se and Pd are 2.1 and 2.2 a.u., respectively. We used 19 × 19 × 1 k-point Monkhorst-Pack mesh for electronic band structure calculations.

Based on the self-consistent converged electronic structure calculations, we have employed the eigenenergies on a very dense nonshifted 8000 k-point mesh in the full Brillouin zone (BZ). Thermoelectrical transport properties were calculated by solving the Boltzmann transport equations within the rigid band (RBA) and constant relaxation-time approximations (CRTA) as implemented in the BoltzTraP software^56^, which neglects the weak energy dependence of relaxation time but retains some temperature and doping dependence^57^. This CRTA approach has been tested earlier and found to work quite well in calculating the Seebeck coefficient in a variety of thermoelectric materials even for materials with highly anisotropic crystal axes^58–61^. A comprehensive description of the Boltzmann transport theory in the relaxation time approximation can be found elsewhere^23^. A brief summary of formalism used in this work is provided below^62^. The energy projected transport distribution (TD) tensor is defined as

3 $${\sigma }_{\alpha \beta }(\varepsilon )=\frac{{e}^{2}}{N}\sum _{i,k}{\tau }_{i,k}{v}_{\alpha }(i,k){v}_{\beta }(i,k)\delta (\varepsilon -{\varepsilon }_{i,k})$$

where group velocity ${v}_{\alpha }(i,k)=\frac{1}{\hslash }\frac{\partial {\varepsilon }_{i,k}}{\partial {k}_{\alpha }}$, *N* is the number of k-points sampled, *τ*_*i*, *k*_ is the band index *i* and wave vector k dependent relaxation time, *α* and *β* are the Cartesian indices, and *e* is the electron charge. Then the electrical conductivity and Seebeck coefficient as a function of temperation *T* and chemical potential *μ*, can be written as

4 $${\sigma }_{\alpha \beta }(T,\mu )=\frac{1}{{\rm{\Omega }}}\int {\sigma }_{\alpha \beta }(\varepsilon )[-\frac{\partial {f}_{0}(T,\varepsilon ,\mu )}{\partial \varepsilon }]d\varepsilon $$

5 $${S}_{\alpha \beta }(T,\mu )=\frac{1}{{e}^{2}T{\rm{\Omega }}}{\sigma }_{\alpha \beta }(T,\mu )\int {\sigma }_{\alpha \beta }(\varepsilon )(\varepsilon -\mu )\times [-\frac{\partial {f}_{0}(T,\varepsilon ,\mu )}{\partial \varepsilon }]d\varepsilon $$

where Ω is the volume of unit cell and *f*_0_ is the Fermi-Dirac distribution function. Thus, by using the CRTA, *τ* is exactly cancelled out in Equation 5. From the above calculations we can obtain the Seebeck coefficient *S* and the electrical conductivity over relaxation time (*σ*/*τ*) as well. The electronic thermal conductivity *k*_*e*_ is calculated using the Wiedemann-Franz law, *k*_*e*_ = *L* *σT*, where *L* is the Lorenz number. In our calculations we use *L* = 2.4 × 10^−8^ *J*^2^ *K*^−2^ *C*^−2^ ^63^.

To confirm the dynamic stability of the PdSe_2_ monolayer, we have calculated the phonon spectrum using a finite displacement method implemented the Phonopy code interfaced with the VASP code^50,64^. At the same time the second-order harmonic IFCs of monolayer PdSe_2_ and third order anharmonic IFCs were calculated using a 4 × 4 × 1 supercell and a 3 × 3 × 1 supercell with Γ point, respectively. Based on an adaptive smearing approach to the conservation of energy^65^ and with an iterative solution method^66^, we then solved the phonon Boltzmann transport equation using ShengBTE^67^.
